# 

**Published:** 1897-12

**Authors:** 


					﻿TABLB■ OF-COKTAJNTS QN XiAST A Dy.EWS1J© FAG£;'■ *
VouXIHa
jMCCEMBEr; 189?.^
ïîo. 6.
TEXAS
Medical Journal.
’ < .^.i>iscR7^ro?i; ^i7oo-: a;¥ear *• i N’*AbvAN’efo;.;/
PUBEÏ5BlEI)AT AlfsïIK. TÎKfÂ8?'BŸ i
Mòrtìban Ä Patr<jbil<ì<S%. jiútTÚÍVR	N<-w
: -Beh terM ’a t Ib&i PÒat<»Ìtfaé at. A nati n. 'Í^X5*s, aà Hécond-e I Wys .Mal 1 M àtwr. ■
E. E. DANIEL: M. D.-. & S. E. HUDSON, M. D.v
AjSl> P^QPÜIEtÔRS'.
				

